# Influence of Hydrodynamic Forces on Electroactive Bacterial Adhesion in Microbial Fuel Cell Anodes

**DOI:** 10.3390/bioengineering10121380

**Published:** 2023-11-30

**Authors:** Alexiane Godain, Timothy M. Vogel, Pascal Fongarland, Naoufel Haddour

**Affiliations:** 1 Ecole Centrale de Lyon, INSA Lyon, Univ Lyon, Université Claude Bernard Lyon 1, CNRS, Ampère, UMR5005, 69130 Ecully, France; 2 Laboratoire d’Ecologie Microbienne, Universite Claude Bernard Lyon 1, UMR CNRS 5557, UMR INRAE 1418, VetAgro Sup, 69622 Villeurbanne, France; vogel@univ-lyon1.fr; 3 CPE-Lyon, CP2M, Universite Claude Bernard Lyon 1, CNRS, UMR 5128, 69616 Villeurbanne, France; pascal.fongarland@univ-lyon1.fr

**Keywords:** microbial fuel cells, shear stress, electroactive bacteria, anodic biofilms, bacterial adhesion

## Abstract

This investigation examined the role of shear stress on the dynamic development of microbial communities within anodic biofilms in single-chamber microbial fuel cells (MFCs). Bacterial attachment to surfaces, often regarded as a crucial step in biofilm formation, may significantly contribute to the selection of electroactive bacteria (EAB). It is well established that hydrodynamic forces, particularly shear forces, have a profound influence on bacterial adhesion. This study postulates that shear stress could select EAB on the anode during the adhesion phase by detaching non-EAB. To examine this hypothesis, MFC reactors equipped with a shear stress chamber were constructed, creating specific shear stress on the anode. The progression of adhesion under various shear stress conditions (1, 10, and 50 mPa) was compared with a control MFC lacking shear stress. The structure of the microbial community was assessed using 16S rRNA gene (rrs) sequencing, and the percentage of biofilm coverage was analyzed using fluorescence microscopy. The results indicate a significant impact of shear stress on the relative abundance of specific EAB, such as *Geobacter*, which was higher (up to 30%) under high shear stress than under low shear stress (1%). Furthermore, it was noted that shear stress decreased the percentage of biofilm coverage on the anodic surface, suggesting that the increase in the relative abundance of specific EAB occurs through the detachment of other bacteria. These results offer insights into bacterial competition during biofilm formation and propose that shear stress could be utilized to select specific EAB to enhance the electroactivity of anodic biofilms. However, additional investigations are warranted to further explore the effects of shear stress on mature biofilms.

## 1. Introduction

Microbial fuel cell (MFC) technology employs electroactive bacteria (EAB) as biocatalysts to generate electricity through the oxidation of organic compounds. This technology has various applications under development, such as sustainable energy production for wastewater treatment and biosensors for monitoring dissolved organic carbon and toxic pollutants [1,2]. The selection of EAB during biofilm formation on the anode is critical for enhancing MFC performance. Multiple strategies have been reported to improve EAB enrichment by exploiting factors that influence biofilm formation, including substrate, temperature, pH, flow rate, anode potential, and anode surface properties [3,4,5,6]. These factors have been shown to play a crucial role in shaping biofilm structure during five different stages of growth: reversible adhesion, irreversible adhesion, microcolony formation, maturation, and dispersion [7]. Godain et al. highlighted the significance of external resistance on the dynamic evolution of these communities, suggesting that strategies inhibiting non-specific electroactive bacteria during initial biofilm formation stages could enhance electricity production [8]. In both natural environments and industrial reactors, microbial biofilms are influenced by various factors, including hydrodynamic forces. Recent studies, such as Hogan et al., emphasize the interplay between bacterial transport and adhesivity, highlighting the significant role of gravitational sedimentation in bacterial adhesion, especially under flow conditions [9]. Indeed, the process of bacterial adhesion is influenced by many factors. A comprehensive review by Zheng et al. summarized the influences of surface charge, surface wettability, roughness, topography, stiffness, and combination of properties on bacterial adhesion, emphasizing the importance of understanding these interactions in various applications [10].

Limited research has been conducted on the effects of shear stress on EAB selection, especially during bacterial adhesion stage, and MFC performance [11]. In their recent study, Nguyen et al. utilized a high-throughput microfluidic platform to investigate the combined effects of antibiotic concentration and fluid shear stress on biofilms. Their findings underscored the nuanced responses of biofilms to these factors, depending on bacterial species and environmental conditions, highlighting the pivotal role of shear stress in directing biofilm evolution in bioelectrochemical systems [12]. Pham et al. found that applying a shear rate of 120 s^−1^ (approximately 120 mPa) resulted in increased power production of MFCs compared to a shear rate of 0.3 s^−1^ (approximately 0.3 mPa) [11]. The results show that the thickness and density of the anodic biofilms were higher under high shear stress than under low shear stress. Thus, shear stress could play a critical role in optimizing biofilm development and enhancing MFC performance. In studies focusing on bacterial community composition, Rochex et al. reported a decrease in diversity after one week when the shear stress increased from 55 to 270 mPa [13]. However, Fang et al. found the opposite result: bacterial diversity increased when the shear stress increased from 2.79 to 21.2 mPa [14]. Both studies demonstrated that shear stress could significantly affect bacterial community composition, which in turn could influence the efficiency and stability of MFCs. Additionally, some studies have used the Reynolds number as an indicator of rotation speed and hydrodynamic force. Oliveira et al. and Ajayi et al. showed increased electricity production at higher Reynolds numbers and suggested a positive relationship between shear stress and MFC performance [15,16].

Despite these insights, the mechanisms underlying the effects of shear stress on bacterial adhesion, biofilm development, and community composition in MFCs remain elusive. This knowledge gap requires further research to elucidate the role of shear stress in EAB selection on the anode to improve strategies to enhance anodic biofilm electroactivity and MFC performance. In this study, MFC reactors equipped with a shear stress chamber were designed and built to create specific shear stress conditions on the anode. The adhesion dynamics under varying shear stress conditions (1, 10, and 50 mPa) were compared with a conventional MFC without shear stress as the control. The microbial community structure was explored using 16S rRNA gene (rrs) sequencing and biofilm coverage was analyzed through fluorescence microscopy.

## 2. Materials and Methods

### 2.1. MFC Setup under Static (No Flow) Conditions

The selection of different configurations for flow and static conditions was a deliberate and strategic decision driven by the inherent requirements of each experimental setup. In the flow condition experiments, the small volume of the chamber limited the number of bacteria able to interact with the electrode surface, which posed a challenge for studying bacterial adhesion effectively. To overcome this limitation and to ensure a comprehensive investigation of bacterial adhesion, a conventional bottle configuration was employed for the static condition experiments. This setup provided a larger interaction volume, allowing for a more substantial bacterial presence and enabling a more accurate observation of the adhesion phase of biofilm formation under static conditions. This approach ensured that each condition was optimally replicated in the laboratory, allowing for accurate assessment and comparison of bacteria adhesion under these distinct environmental scenarios. For static conditions, single-chamber batch MFCs were set up using 250 mL Wheaton bottles in the laboratory at ambient temperature (Figure S1). The anode material was sourced from a carbon cloth, segmented into 25 sections, each measuring 0.5 × 8 cm from original dimensions of 10 × 15 cm (Figure S2). The air cathode was prepared with a PTFE coating and a 5% platinum catalyst, following the procedure outlined by Cheng et al. [17]. Each MFC was filled with 250 mL of primary effluent and 1 g of dehydrated sludge obtained from a municipal wastewater treatment plant in Lyon, France, and then fed with 1 g·L^−1^ of sodium acetate. The experiment was conducted in duplicate (a and b). The anode was connected to the cathode through an external resistance of 1000 ohms (MFC-1000_a, MFC-1000_b). Two MFCs were initiated without external resistance and served as negative controls (MFC-CT_a and MFC-CT_b). The MFC voltage was recorded every 5 min with a precision of 1 µV. At each time point (0.5, 1, 4, and 8 h), two anodic samples (0.5 × 8 cm) were collected from each MFC. One portion of the anode (0.5 × 6 cm) was used for DNA extraction, while the other portion (0.5 × 2 cm) was utilized for microscopic observations. In this study, the focus was primarily on the adhesion stage of biofilm formation under flow conditions. Static conditions, while acknowledged, were not experimentally compared to flow conditions due to the distinct setup required. Specifically, in the static setup, a conventional bottle configuration was used, differing significantly from the flow configuration where the channel volume is minimal. The static condition was considered during the discussion of our results. This approach allowed for a broader interpretation of biofilm adhesion dynamics across different environmental setups. It is critical to understand that the distinct experimental setups for static and flow conditions led to this methodological decision.

### 2.2. MFC Setup under Flow Conditions

#### 2.2.1. Reactor Design

**Figure 1:**
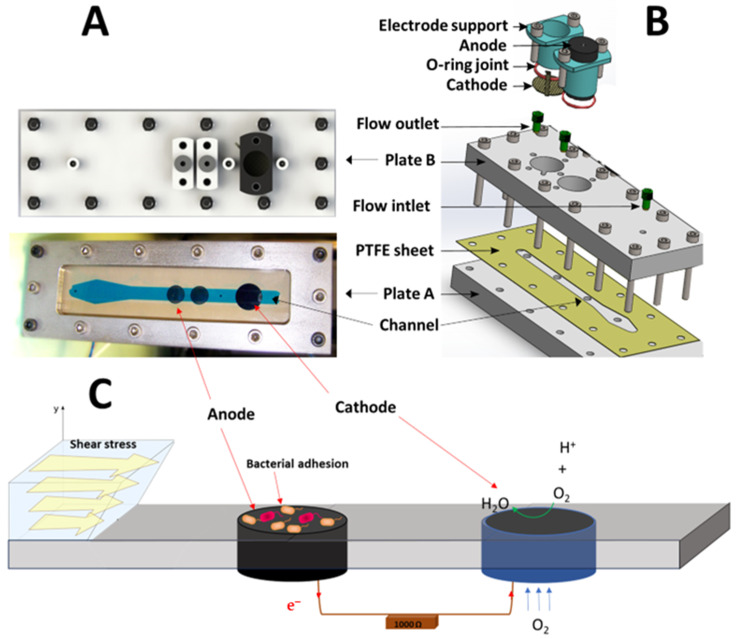
(**A**) Photo, (**B**) schematic, and (**C**) operating principle of MFC configuration with a shear stress flow chamber.

Three specific MFCs were designed and built to control the shear stress applied on the anodic surface. The configuration of the shear stress flow chamber was chosen for improved quality control and the homogeneity of the shear stress field. Each reactor (Figure 1) consisted of (i) one upper PMMA plate B (500 mm × 240 mm × 20 mm), (ii) one bottom PMMA plate A (500 mm × 240 mm × 20 mm) with different openings for fluid to enter and exit and for the electrodes, and (iii) one hollowed PTFE plate shim (500 mm × 240 mm × 0.5 mm) to channel the fluid flow. The plates were held together by 70 screws and bolts. The electrodes were a graphite rod (ø24 × 50 mm) for the anode and an air cathode (ø24 mm). A plastic piece held each electrode and was easily removed by removing the two screws. An O-ring maintained the seal around the electrode. The hydrodynamic characterization of this configuration was previously carried out by Lorthois et al. [18,19] and the forces applied on a particle for this configuration were provided by O’Neil [20]. The thickness of the channel was small (0.5 mm) in comparison to the width of the channel (10 mm). In such a channel, the flow is theoretically a two-dimensional Poiseuille flow. A diverging–converging channel at the entrance of the fluid ensures a homogenous shear stress in the channel. The shear stress was uniform except near a confined part close to the channel side walls. In this configuration, the shear stress can be expressed by the following Equation (1) [18,19]:

*τ* = 3µQ/4w2t (1)

where *τ* is the shear stress in Pa, µ is the dynamic viscosity in Pa·s, Q is the flow rate in m^3^·s^−1^, w is the half width of the channel in meters, and t is the half thickness of the channel in meters. The drag force D applied on an isolated bacterium on a surface can be expressed as a function of the shear stress by Equation (2) [18,19]:

D = 32*τ*r^2^ (2)

with r as the bacterial radius in m (about 1 × 10^−6^ m).

#### 2.2.2. Experimental Setup

MFCs were started under three different shear stresses (1, 10, and 50 mPa) with hydrodynamic characteristics, as presented in Table 1. An external resistance of 1000 ohms was used for all channels. The voltage of each channel was recorded every 5 min. A phosphate buffer was used as a medium amended with 1 g·L^−1^ of sodium acetate (pH 7.4, 7 mS·cm^−2^). This solution contained 4 g·L^−1^ of dehydrated sludge sourced from a municipal wastewater treatment plant located in Lyon, France. The pumps were syringe pumps to ensure a homogenous fluid flow at 22 °C as a function of time. A head loss was added at the outlet to ensure an equal fluid flow in each channel.

### 2.3. Microscopic Observations

The anodic biofilms were observed by fluorescence microscopy. The samples were labeled using a solution of SYBR green diluted to 1/50. Five µL of SYBR green was mixed in 2 mL of sterile NaCl 0.8%. Then, 200 µL was deposed on each sample. Samples were incubated for 15 min in the dark before observations. Four images were taken per sample using a z-stack of one image per 10 µm and a focus ×200. The image size was 1388 × 1040 pixels with a resolution of 150 dpi. The biofilm coverage percentage was determined using the ImageJ software (v.1.52s) [21]. In order to remove the out-of-focus signal recorded for each individual image, different filters were applied. First, a 3D median filter was applied, then the background was subtracted with a rolling ball radius of 50 pixels. Then, the maximal intensity z-projection was applied. Finally, a threshold was applied for each channel. The number of white pixels of each channel divided by the total number of pixels was calculated and used as the coverage percentage.

### 2.4. 16S rRNA Gene Sequencing

For each sample (0.5 × 6 cm^2^), DNA was extracted using the Nucleospin Soil (Macherey-Nagel, Duren, Germany) The solution SL1 was used for the cell lysis and DNA was eluted using 2 × 25 µL of the elution solution. Then, the region V3-V4 of the gene that codes for the 16S rRNA was amplified using Platinum Taq DNA Polymerase of ThermoFisher Scientific, Dardilly, France. The forward primer sequence was: 5′ TCGTCGGCAGCGTCAGATGTGTATAAGAGACAGCCTACGGGNGGCWGCAG 3′, and the reverse primer sequence was: 5′GTCTCGTGGGCTCGGAGATGTGTATAAGAGACAGGACTACHVGGGTATCTAATCC. The PCR program was 95 °C for 3 min, 25 cycles of 95 °C for 30 s, 55 °C for 30 s and 72 °C for 30 s, and then a final step of 72 °C for 5 min. The resulting amplicon size was about 550 bp. Then, library preparation was performed with regard to the Illumina protocol. The amplicons were sequenced by a paired-end MiSeq sequencing using the technology V3 of Illumina with 2 × 300 cycles. The adapter sequences were removed using internal Illumina software at the end of the sequencing (forward overhang 5′TCGTCGGCAGCGTCAGATGTGTATAAGAGACAG and reverse overhang 5′GTCTCGTGGGCTCGGAGATGTGTATAAGAGACAG). To remove the low base quality at the end of the read 2, the read 2 sequences were cut at the 200th base using the command—fastq_filter and the option—fastq_trunclen of USEARCH [22]. Then, Pandaseq was used to link read 1 and 2 using a minimal and maximal length of 430 and 490 bp, a minimal and maximal overlap of 30 and 60 bp, and a quality threshold of 0.9 [23]. The mean and the median of the paired sequence percentage were 90.59% (standard error 6.00%) and 90.62%, respectively. The repartition of the sequence number after paired-end assembling is shown in Figure S3 (mean = 51,446 ± 18,240 sequences per sample). Other parameters were left at default values. The resulting sequences (length mean: 465 bp) were annotated using an RDP classifier in the RDP database and an assignment confidence cutoff of 0.8 [24]. In the bacterial community analysis, family rank was chosen as a good compromise between the precision of the taxonomic rank and the annotation percentage. Based on the research conducted by C. Koch et al., which cataloged 69 species capable of anodic electron transfer, belonging to 37 genera [25]. The bacterial genera considered as potential EAB in this study were *Geobacter*, *Arcobacter*, *Desulfovibrio*, *Clostridium*, *Pseudomonas*, *Shewanella*, *Streptomyces*, *Bacillus*, *Aeromonas*, *Rhodoferax*, and *Escherichia*. Two bacterial communities were particularly targeted: nonspecific adapted EAB (composed of Pseudomonadaceae, Comamonadaceae, and Moraxellaceae), and specific adapted EAB (composed of Geobacteraceae, Desulfuromonadaceae, Clostridiaceae, and Desulfovibrionaceae).

### 2.5. Statistical Analysis

A diversity analysis was conducted at the genus level using R software (v.R 3.5.3) and the R package vegan. Then, 200 subsamples of 10,000 sequences were repetitively taken in each sample, in order to compare the diversity between samples. The means for the number of genera and the Shannon index were calculated for each sample from these 200 subsamples. Statistical tests were conducted using R software too. The normal distribution of data was tested using the function shapiro.test. If the data fit a normal distribution, parametric tests (t.test) were used. If the data did not fit a normal distribution, non-parametric tests were used (wilcox.test). In order to compare the adhesion rates, linear models using the R function lm were built and compared. Linear models were of type y = Ax. The coefficients A were compared using Student’s *t*-test. This test was calculated using the following Equation (3):

*T* = |A1−A2|/√(Sd1^2^ + Sd2^2^) (3)

where *T* is the Student’s *t*-test, A1 and A2 are the coefficients of the model 1 and 2; Sd1 and Sd2 are the standard errors of A1 and A2.

## 3. Results

### 3.1. Anodic Bacteria Adhesion under Shear Stress Conditions

**Figure 2:**
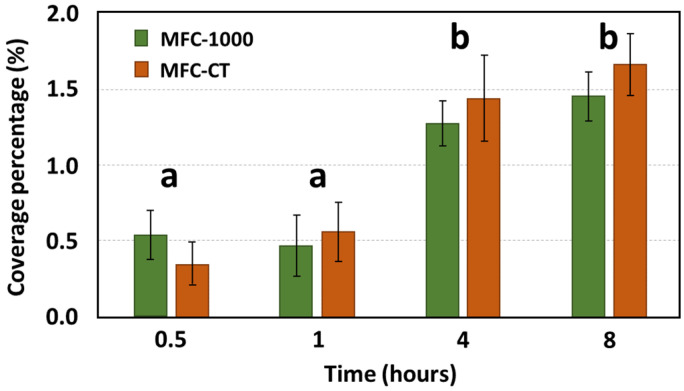
Anodic coverage percentage as a function of time under static conditions. The coverage percentage was calculated from eight fluorescence microscopic images taken at a focus ×200. The error bars represent the error deviation. The letters correspond to statistical group after a Wilcoxon test at a *p*-value < 0.05.

**Figure 3:**
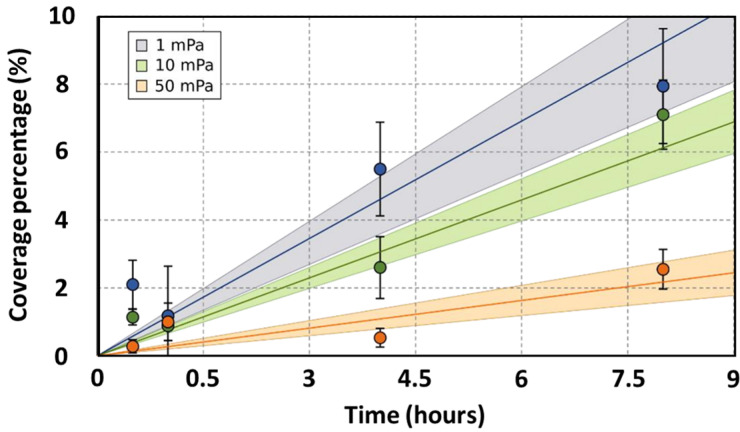
Coverage kinetic models. The models are linear and of the type y = Ax. Three models were built corresponding to three shear stress conditions: 1 mPa (blue), 10 mPa (green), and 50 mPa (red). The lines represent the linear models. The points represent the experimental data. The coverage percentage was calculated from four fluorescence microscopic images taken at ×200. The error bars are the error deviations. The colored area is the confidence interval at 90% of predicted values.

Microscopic evaluations during the initial hours of bacterial adhesion were employed to ascertain the coverage percentage of the anode. Under static conditions, biofilm coverage between MFC-1000 and MFC-CT exhibited no significant disparity (Figure 2). After an initial 30 min period, coverage percentages for MFC-1000 and MFC-CT were recorded as 0.54 ± 0.16% and 0.35 ± 0.20%, respectively. The subsequent hour showed no notable variations. However, after 4 h, coverage percentages rose to 1.27 ± 0.15% for MFC-1000 and 1.44 ± 0.28% for MFC-CT. By the 8-hour mark, the percentages had further increased to 1.45 ± 0.16% and 1.66 ± 0.20% for MFC-1000 and MFC-CT, respectively. Under flow conditions, biofilm coverage was assessed at three distinct shear stresses: 1, 10, and 50 mPa. At the 30 min mark, coverage percentages were 2.37 ± 0.71%, 1.14 ± 0.23%, and 0.53 ± 0.19% for shear stresses of 1, 10, and 50 mPa, respectively. After 8 h, a pronounced difference was observed among the conditions, with coverage percentages recorded as 7.50 ± 1.69%, 6.38 ± 1.02%, and 2.15 ± 0.59% for shear stresses of 1, 10, and 50 mPa, respectively. To facilitate a comparative analysis of coverage rates across varying shear stresses, linear models of the form y = Ax were constructed (Figure 3), where ‘y’ represents the coverage percentage, ‘x’ denotes time, and ‘A’ is the linear coefficient. These linear models accounted for a substantial proportion of the coverage variability, with an R^2^ value exceeding 0.7, and were statistically significant based on their *p*-values (<10^−4^). The coefficients A were 1.11 ± 0.12%, 0.77 ± 0.06%, and 0.27 ± 0.04% for 1, 10, and 50 mPa, respectively. A Student’s *t*-test was performed between the coefficients A, revealing a significant difference between coefficients at a *p*-value < 0.01. As a result, the adhesion rates under shear stresses of 10 mPa and 50 mPa were 1.44 h and 4.11 times slower, respectively, than under a shear stress of 1 mPa. Upon comparing the static and flow conditions, a distinct difference in bacterial adhesion dynamics becomes evident. While static conditions yielded a steady, albeit modest, increase in anodic coverage over time, the flow conditions, particularly at lower shear stresses, demonstrated a markedly higher bacterial adhesion rate. For instance, at a shear stress of 1 mPa, the anodic coverage after 8 h was significantly greater than that observed under static conditions. This highlights the role of even minimal fluid movement in enhancing bacterial adhesion compared to completely static environments. Conversely, at higher shear stresses (10 mPa and 50 mPa), the rate of biofilm formation was reduced, suggesting an optimal threshold of shear stress that promotes the bacterial adhesion rate.

### 3.2. Selection of Bacteria under Shear Stress Conditions

To determine whether shear stress influences the selection of EAB, the bacterial composition of the biofilms was analyzed. Under static conditions, no significant difference in bacterial composition was observed between MFC-1000 and MFC-CT (Figure S4). Throughout the observation period, from 30 min to 8 h, no temporal variation in specific EAB was detected. The average relative abundance of specific EAB remained consistent at 1.02 ± 0.44% during this timeframe, indicating no preferential selection of specific EAB during bacterial adhesion. Conversely, the relative abundance of nonspecific EAB exhibited a time-dependent increase, rising from 61.16 ± 17.26% at the 30 min mark to 79.40 ± 12.71% by the 8 h mark. This trend suggests a selection bias towards nonspecific EAB under static conditions, supported by a statistically significant *p*-value (<0.05).

**Figure 4:**
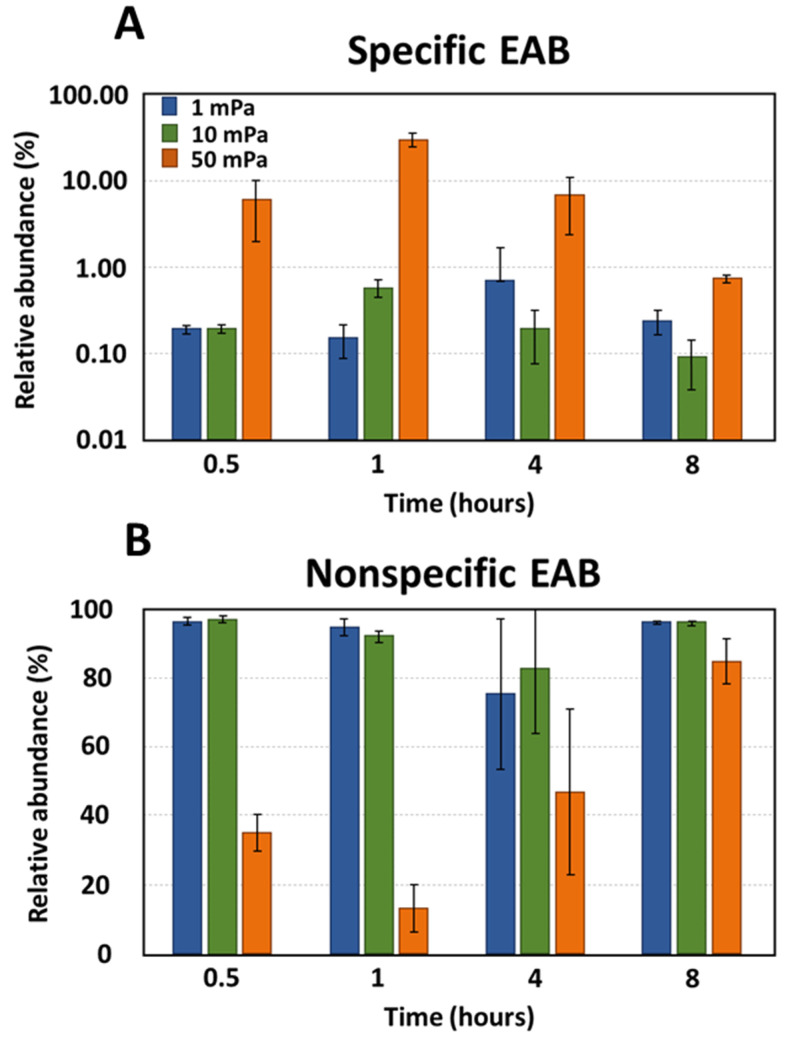
The relative abundance of (**A**) specific EAB and (**B**) non-specific EAB under shear stress conditions. The error bars represent the standard deviations.

**Figure 5:**
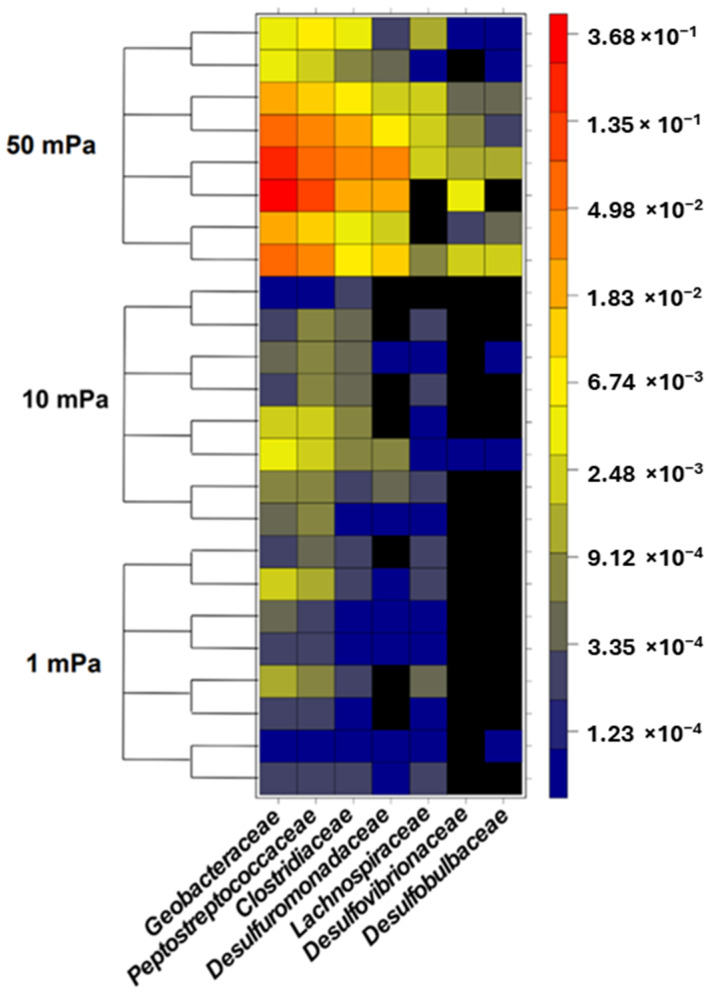
Relative abundance of each family considered as specific EAB present in the anodic biofilms.

The bacterial composition of biofilms was also assessed under flow conditions that induced shear stresses of 1, 10, and 50 mPa (Figure 4). Under shear stresses of 1 mPa and 10 mPa, the relative abundance of specific EAB remained consistent and ranged between 0.09% and 0.69%. Therefore, this indicated no preferential selection of specific EAB during bacterial adhesion at these shear levels. However, at a shear stress of 50 mPa, a significant deviation in the relative abundance of specific EAB was observed compared to both 1 mPa (*p*-value < 10^−3^) and 10 mPa (*p*-value < 10^−4^). On average, the presence of specific EAB was amplified by factors of 61 and 38 under a shear stress of 50 mPa compared to shear stresses of 1 mPa and 10 mPa, respectively. This increased shear stress evidently favored the selection of specific EAB. Bacterial families such as *Geobacteriaceae*, *Desulfuromonodaceae*, *Peptostreptococcaceae*, and *Clostridiaceae* emerged as the predominant bacteria during the adhesion phase under a shear stress of 50 mPa (Figure 5). Notably, this selection was most pronounced within the initial hour, with the relative abundance surging from 6.13% at the 30 min mark to 30.14% after 1 h. However, this abundance subsequently declined to 6.79% after 4 h and further to 0.74% after 8 h. By the 8 h interval, the relative abundance of specific EAB under a shear stress of 50 mPa was indistinguishable from that observed under shear stresses of 1 mPa and 10 mPa. Under shear stresses of 1 mPa and 10 mPa, the relative abundance of non-specific EAB remained relatively constant across all observed time points with values oscillating between 75.39% and 96.99%. In contrast, at a shear stress of 50 mPa, a significant reduction in the relative abundance of non-specific EAB was noted compared to the values observed at 1 mPa (*p*-value < 0.01, mean difference = 50.39%) and 10 mPa (*p*-value < 0.01, mean difference = 50.37%). This decline in non-specific EAB was most pronounced during the initial hours with the relative abundance diminishing from 35.23 ± 5.23% to 12.96 ± 6.96%. However, after a 4 h duration, there was a resurgence in the relative abundance, reaching 46.83 ± 24.36%. By the 8 h mark, the relative abundance of non-specific bacteria converged across all shear stress conditions, registering at 96.0 ± 5.05%, 95.94 ± 0.64%, and 84.71 ± 6.71% for shear stresses of 1 mPa, 10 mPa, and 50 mPa, respectively.

### 3.3. Evolution of Bacterial Diversity

**Figure 6:**
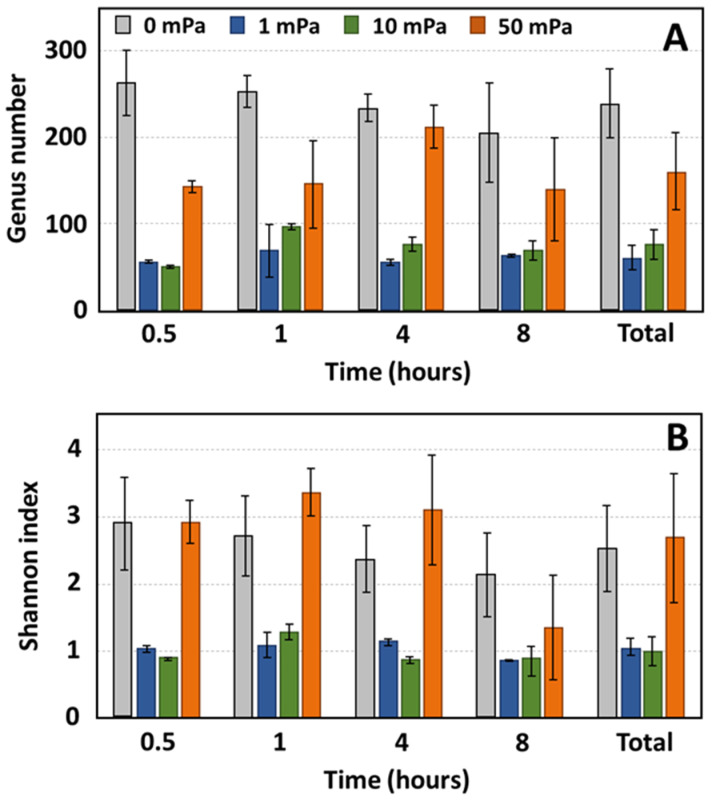
Evolution of diversity as a function of time for shear stresses of 1, 10, and 50 mPa. The genus number ((**A**): richness) and the Shannon index ((**B**): richness and evenness) are represented; ‘0 mPa’ refers to the static conditions MFC-1000 and “Total” represents the aggregated data from samples collected after an 8 h experimental period, specifically between 10 and 11 h, marking the conclusion of the observation period.

Bacterial diversity was assessed for each sample across both static and flow conditions (Figure 6). Under static conditions, the anode exhibited a greater genus diversity compared to any of the shear stress environments. Specifically, 262 genera were identified after 30 min, and this number decreased to 204 by the 8 h mark under static conditions. In contrast, under shear stresses of 1 mPa and 10 mPa, the genus number was notably reduced, with 56 and 51 genera identified after 30 min, respectively. This count marginally increased to 63 and 69 genera after 8 h for shear stresses of 1 mPa and 10 mPa, respectively. At a shear stress of 50 mPa, the genus number surpassed that of the other shear stress conditions, albeit remaining lower than the static condition, with 142 genera registered at the 30 min mark and 139 genera after 8 h. The Shannon diversity index values after 8 h were 2.12, 0.86, 0.90, and 1.35 for shear stresses of 0, 1, 10, and 50 mPa, respectively.

## 4. Discussion

### 4.1. Complex Effect of Shear Stress on Bacterial Adhesion

The present investigation demonstrated a decline in the coverage rate with increasing shear stress. The coverage percentage adhered to a linear model represented by y = Ax, where the coefficient ‘A’ was 1.11 ± 0.12% for 1 mPa, 0.77 ± 0.06% for 10 mPa, and 0.27 ± 0.04% for 50 mPa. Consequently, after an 8 h duration, the coverage percentages were 7.50 ± 1.69%, 6.38 ± 1.02%, and 2.15 ± 0.59% for shear stresses of 1, 10, and 50 mPa, respectively. Unlike detachment experiments, where bacterial adhesion occurs in the absence of shear stress [26,27], the process of adhesion in this context is not solely governed by adsorption forces. Flow velocity modifies the probability of bacterial surface contact. The influence of shear stress becomes even more pronounced when considering bacterial load. The corresponding flow rates for shear stresses of 1, 10, and 50 mPa were 0.75, 7.5, and 37.5 mL·h^−1^, respectively. After 8 h, the cumulative volume through the reactors amounted to 6, 60, and 300 mL for shear stresses of 1, 10, and 50 mPa, respectively. This increased volume enhances bacterial surface contact. Sedimentation forces, depending on the bacterial residence time within the reactor, can also modulate adhesion. Residence times within the reactor were approximately 176.0, 17.6, and 3.5 min for a reactor volume of 2.2 mL. We used the Stokes Equation (4):

*v_s_* = 2r^2^gΔρ/9µ (4)

where *v_s_* is the sedimentation velocity, r is the particle radius estimated for a bacteria to 0.5 µm, g is the gravitational acceleration of 9.81 m·s^−2^, Δρ is the volume density difference between the fluid (estimated to 1000 kg·m^−3^) and the bacteria (estimated to 1100 kg·m^−3^ [28]), and µ is the dynamic viscosity of the fluid (10^−3^ kg·m^−1^·s^−1^). The characteristic sedimentation time for a distance of 500 µm (the channel height) is 152.9 min. The sedimentation favors the adhesion by increasing the contact probability only under a shear stress of 1 mPa but not under the other shear stresses, where the residence time is too short in comparison to the characteristic sedimentation time. Other forces, such as the shear gradient lift force or the Magnus force, could modify the contact probability, but in the case where the particle is not buoyant, these forces are considered negligible [29,30].

### 4.2. Specificity of Electroactive Bacteria Adhesion

The present study observed that elevated shear stress, specifically at 50 mPa, favored the selection of specific EAB, reaching a relative abundance of up to 30% after 1 h. In contrast, shear stresses of 1 and 10 mPa resulted in a relative abundance of less than 1% for specific EAB. Notably, the Geobacteriaceae family emerged as the predominant selection. This family had members that exhibit superior surface adhesion capabilities compared to other bacterial species. Such findings can be attributed to the natural environment where specific EAB, such as *Geobacter*, utilize insoluble metal oxide particles as electron acceptors. To thrive and proliferate under these conditions, this bacterium must exhibit competitive adhesion to these metal oxides. This enhanced adhesion capability might be influenced by factors such as (i) hydrophilic/hydrophobic membrane characteristics, (ii) membrane potential, and/or (iii) specific membrane proteins. The extended DLVO theory encompasses three forces: the Van der Waals force, the electrostatic force, and the Lewis acid/base force. While the Van der Waals force remains independent of bacterial membrane properties, the electrostatic force is influenced by the bacterial membrane potential, and the Lewis acid/base force is dependent on the hydrophilic membrane characteristics of bacteria. Consequently, these forces might contribute to superior adhesion of *Geobacter*. Additionally, the production of specific membrane proteins, such as nanowires, could further enhance adhesion, enabling metal-reducing bacteria to firmly adhere to surfaces. However, after 8 h, no significant difference in the relative abundance of specific EAB was observed between high and low shear stress conditions. This phenomenon might be attributed to (i) initial bacterial populations creating a surface more favorable to the adhesion of subsequent bacteria, and/or (ii) the accelerated growth rate of non-specific bacteria compared to specific EAB. For instance, considering two bacterial species—one with a doubling time of 1 h and another with a doubling time of 10 h—the concentration of the former was amplified by a factor of 256, while the latter only achieved a 1.1-fold increase after 8 h.

### 4.3. Potential Impact on MFC Performances

Shear stress may serve as a mechanism for enhancing current density in MFCs by promoting the prevalence of specific electroactive bacteria within anodic biofilms. The present investigation revealed that after a duration of one hour under a shear stress of 50 mPa, the proportion of specific EAB reached 30%, whereas it remained below 1% under shear stresses of 1 and 10 mPa. Conversely, elevated shear stress was associated with a reduction in biofilm coverage percentage. Several studies have posited a correlation between power density and biofilm coverage [30,31]. For instance, Li et al. identified a relationship between current density and biomass density on the anode [32]. Therefore, while a high shear stress might promote the presence of specific EAB, it could be detrimental if it impedes the formation of a densely covered biofilm on the anode. Extended research is warranted to elucidate the long-term effects of shear stress. Moreover, the proportion of specific EAB observed after 8 h did not exhibit significant variations across different shear stress conditions. A prolonged examination of biofilm development is essential to validate the potential selection of specific EAB under increased shear stress.

## 5. Conclusions

This study thoroughly investigates the influence of shear stress on bacterial adhesion to anodic surfaces in MFCs. Our findings highlight the pivotal role of shear stress, particularly at 50 mPa, in modulating the prevalence of EAB within anodic biofilms. Notably, under the condition of 50 mPa shear stress, there was a significant increase in the relative abundance of specific EAB, with *Geobacteriaceae* emerging as the dominant family. This suggests that EAB, adapted to competitive adhesion in natural environments, might display enhanced adhesion capabilities under selected shear stress conditions. However, it is important to note that while high shear stress favors the adhesion of specific EAB, it concurrently leads to a reduction in the overall biofilm coverage on the anode. This finding is crucial, as several studies have correlated power density with biofilm coverage, indicating a trade-off that necessitates further exploration. Thus, striking a balance between enhancing specific EAB prevalence and maintaining optimal biofilm coverage is critical for maximizing MFC performance. Additionally, our study indicates that the initial positive effects of high shear stress on specific EAB selection diminish over extended periods. Therefore, future research should aim to explore the long-term impact of shear stress on biofilm development and composition. This could involve investigating the benefits of applying high shear forces initially to boost specific EAB presence before transitioning to conditions more conducive to biofilm growth. In summary, this study provides significant insights into the role of shear stress in shaping anodic biofilm development in MFCs. The potential benefits of employing shear stress as a tool for enhancing MFC efficiency are evident, yet a comprehensive understanding of the associated trade-offs and long-term effects is imperative. Such knowledge is essential for the practical application of these findings in the design and operation of MFCs.

## Figures and Tables

**Table 1:** Reynolds number, shear stress, and drag force as a function of the fluid flow.

*τ* (mPa)	Q (mL·h^−1^)	Re	D (pN)
1	0.75	0.021	0.032
10	7.50	0.208	0.320
50	37.50	1.042	1.600

## Data Availability

The data presented in this study are available in Supplementary Materials.

## Supplementary Materials

The following supporting information can be downloaded at: https://www.mdpi.com/article/10.3390/bioengineering10121380/s1, Figure S1: MFC bottle with an air cathode; Figure S2: Schematic of the anodes; Figure S3: Histogram of the repartition of the number of samples in function of the number of sequences; Figure S4: The relative abundance of EAB under static conditions.

## References

[1] Bataillou G., Lee C., Monnier V., Gerges T., Sabac A., Vollaire C., Haddour N. (2022). Cedar Wood—Based Biochar: Properties, Characterization, and Applications as Anodes in Microbial Fuel Cell. Appl. Biochem. Biotechnol..

[2] Haddour N., Azri Y.M. (2023). Recent Advances on Electrochemical Sensors Based on Electroactive Bacterial Systems for Toxicant Monitoring: A Minireview. Electroanalysis.

[3] Prévoteau A., Rabaey K. (2017). Electroactive Biofilms for Sensing: Reflections and Perspectives. ACS Sensors.

[4] Pinck S., Ostormujof L.M., Teychené S., Erable B. (2020). Microfluidic Microbial Bioelectrochemical Systems: An Integrated Investigation Platform for a More Fundamental Understanding of Electroactive Bacterial Biofilms. Microorganisms.

[5] Greenman J., Gajda I., You J., Mendis B.A., Obata O., Pasternak G., Ieropoulos I. (2021). Microbial Fuel Cells and Their Electrified Biofilms. Biofilm.

[6] Paitier A., Haddour N., Gondran C. (2022). Effect of Contact Area and Shape of Anode Current Collectors on Bacterial Community Structure in Microbial Fuel Cells. Molecules.

[7] Stoodley P., Sauer K., Davies D.G., Costerton J.W. (2002). Biofilms as Complex Differentiated Communities. Annu. Rev. Microbiol..

[8] Godain A., Haddour N., Fongarland P. (2022). Bacterial Competition for the Anode Colonization under Different External Resistances in Microbial Fuel Cells. Catalysts.

[9] Hogan K., Compton R.G., Kätelhön E., Ward K.R., Laborda E. (2023). Effect of Gravity on Bacterial Adhesion to Heterogeneous Surfaces. Pathogens.

[10] Zheng S., Bawazir M., Dhall A., Kim H.E., He L., Heo J., Hwang G. (2021). Implication of Surface Properties, Bacterial Motility, and Hydrodynamic Conditions on Bacterial Surface Sensing and Their Initial Adhesion. Front. Bioeng. Biotechnol..

[11] Pham H.T., Boon N., Aelterman P., Clauwaert P., De Schamphelaire L., Van Oostveldt P., Verbeken K., Rabaey K., Verstraete W. (2008). High Shear Enrichment Improves the Performance of the Anodophilic Microbial Consortium in a Microbial Fuel Cell. Microb. Biotechnol..

[12] Nguyen A.V., Shourabi A.Y., Yaghoobi M., Zhang S., Simpson K.W., Abbaspourrad A. (2022). A High-Throughput Integrated Biofilm-on-a-Chip Platform for the Investigation of Combinatory Physicochemical Responses to Chemical and Fluid Shear Stress. PLoS ONE.

[13] Rochex A., Godon J.J., Bernet N., Escudié R. (2008). Role of Shear Stress on Composition, Diversity and Dynamics of Biofilm Bacterial Communities. Water Res..

[14] Fang H., Chen Y., Huang L., He G. (2017). Analysis of Biofilm Bacterial Communities under Different Shear Stresses Using Size-Fractionated Sediment. Sci. Rep..

[15] Oliveira V., Carvalho T., Melo L., Pinto A., Simões M. (2016). Effects of Hydrodynamic Stress and Feed Rate on the Performance of a Microbial Fuel Cell. Environ. Eng. Manag. J..

[16] Ajayi F.F., Kim K.Y., Chae K.J., Choi M.J., Kim I.S. (2010). Effect of Hydrodymamic Force and Prolonged Oxygen Exposure on the Performance of Anodic Biofilm in Microbial Electrolysis Cells. Int. J. Hydrogen Energy.

[17] Cheng S., Liu H., Logan B.E. (2006). Increased Performance of Single-Chamber Microbial Fuel Cells Using an Improved Cathode Structure. Electrochem. Commun..

[18] Lorthois S., Schmitz P., Houi D., Angles-Cano E. (2000). Experimental Study of Fibrin Embolization under Shear Flow. J. Adhes..

[19] Lorthois S., Schmitz P., Anglés-Cano E. (2001). Experimental Study of Fibrin/Fibrin-Specific Molecular Interactions Using a Sphere/Plane Adhesion Model. J. Colloid Interface Sci..

[20] O’Neill M.E. (1968). A Sphere in Contact with a Plane Wall in a Slow Linear Shear Flow. Chem. Eng. Sci..

[21] Schindelin J., Rueden C.T., Hiner M.C., Eliceiri K.W. (2015). The ImageJ Ecosystem: An Open Platform for Biomedical Image Analysis. Mol. Reprod. Dev..

[22] Edgar R.C. (2010). Search and Clustering Orders of Magnitude Faster than BLAST. Bioinformatics.

[23] Massela A., Bartram A.K., Truszkowski J.M., Brown D.G., Neufeld J.D. (2012). PANDAseq: PAired-END Assembler for Illumina Sequences. Gut.

[24] Wang Q., Garrity G.M., Tiedje J.M., Cole J.R. (2007). Naïve Bayesian Classifier for Rapid Assignment of RRNA Sequences into the New Bacterial Taxonomy. Appl. Environ. Microbiol..

[25] Koch C., Harnisch F. (2016). Is There a Specific Ecological Niche for Electroactive Microorganisms?. ChemElectroChem.

[26] Guillemot G., Lorthois S., Schmitz P., Mercier-Bonin M. (2007). Evaluating the Adhesion Force between Saccharomyces Cerevisiae Yeast Cells and Polystyrene from Shear-Flow Induced Detachment Experiments. Chem. Eng. Res. Des..

[27] Duddridge J.E., Kent C.A., Laws J.F. (1982). Effect of Surface Shear Stress on the Attachment of Pseudomonas Fluorescens to Stainless Steel under Defined Flow Conditions. Biotechnol. Bioeng..

[28] Baldwin W.W., Myer R., Powell N., Anderson E., Koch A.L. (1995). Buoyant Density of Escherichia Coli Is Determined Solely by the Osmolarity of the Culture Medium. Arch. Microbiol..

[29] Gutfinger C., Pnueli D., Moldavsky L., Shuster K., Fichman M. (2003). Particle Motion in Simple Shear Flow with Gravity. Aerosol Sci. Technol..

[30] Zhang J., Yan S., Yuan D., Alici G., Nguyen N.T., Ebrahimi Warkiani M., Li W. (2016). Fundamentals and Applications of Inertial Microfluidics: A Review. Lab Chip.

[31] Cornejo J.A., Lopez C., Babanova S., Santoro C., Artyushkova K., Ista L., Schuler A.J., Atanassov P. (2015). Surface Modification for Enhanced Biofilm Formation and Electron Transport in Shewanella Anodes. J. Electrochem. Soc..

[32] Li N., Kakarla R., Min B. (2016). Effect of Influential Factors on Microbial Growth and the Correlation between Current Generation and Biomass in an Air Cathode Microbial Fuel Cell. Int. J. Hydrogen Energy.

