# Supplementary material for: Influence of Hydrodynamic Forces on Electroactive Bacterial Adhesion in Microbial Fuel Cell Anodes
Source: Bioengineering (Basel). 2023 Nov 30;10(12):1380. doi: 10.3390/bioengineering10121380 (PMC10740411; doi:10.3390/bioengineering10121380)
Supplement: Supplementary file 1 [file bioengineering-10-01380-s001.zip › bioengineering-2712571-supplementary.pdf]

Supplementary Materials

Study of bacterial adhesion under flow conditions in microbial fuel cell

Keywords: Microbial fuel cells; shear stress; electroactive bacteria; anodic biofilms; bacterial adhesion.

S1. MFC setup and sampling

MFC bottles MFCs with a "classical" configuration were used (Figure S1). The cathode was an air cathode created by following the procedure of Cheng et al. [1]. It contains one catalytic slide (the inner side) and one diffusion side (the outer side). The catalytic side is composed of carbon powder with 5% platinum powder. The diffusion side is composed of 4 layers of PTFE (Polytetrafluoroethylene).

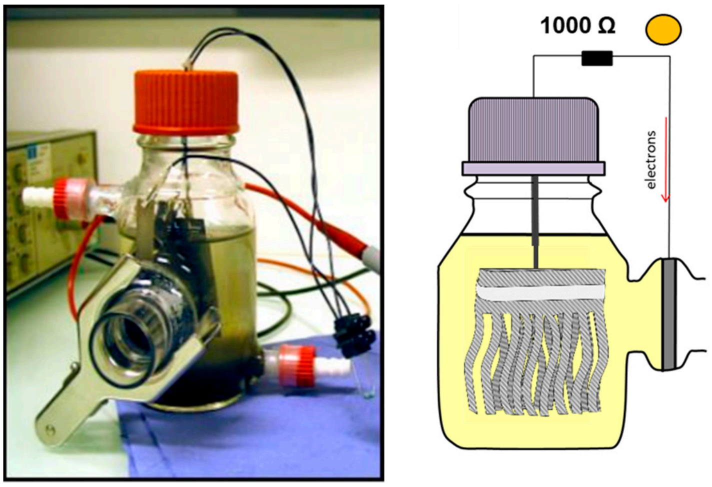

Figure S1. MFC bottle with an air cathode.

The anode consisted of one 10 x 15 cm piece of carbon cloth. The anode was constituted of 25 carbon cloth strips of 80 x 5 mm. A titanium strip was use as the collector.

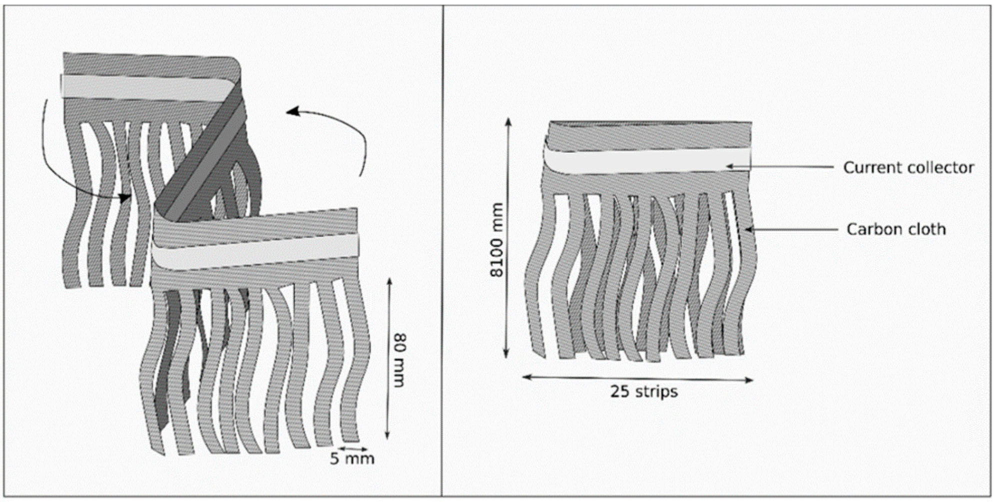

Figure S2. Schematic of the anodes.

The experiment was done in duplicate (a and b) separated by a period of 3 months. 2x4 MFCs were started with different external resistances: 1000 ohms (M-1000-a and M-1000-b), 330 ohms (M-330-a, M-330-b), without resistance (M-0-a and M-0-b) and two with an open circuit simulating an infinite resistant (M-inf-a, M-inf-b) (Figure S6).

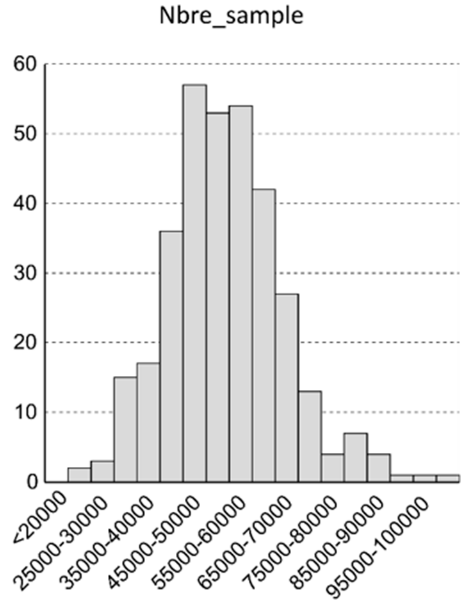

Figure S3. Histogram of the repartition of the number of samples in function of the number of sequences

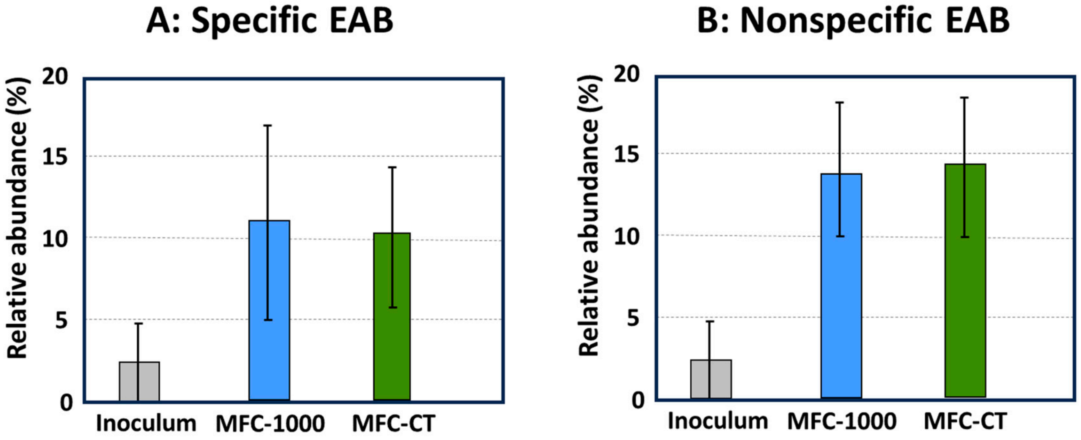

Figure S4. The relative abundance of EAB under static conditions. The error bars represent the standard deviations.

Reference

1. Cheng, S.; Liu, H.; Logan, B.E. Increased Performance of Single-Chamber Microbial Fuel Cells Using an Improved Cathode Structure. *Electrochem. Commun.* **2006**, *8*, 489–494. https://doi.org/10.1016/j.elecom.2006.01.010.
